# Differences in HBV Replication, APOBEC3 Family Expression, and Inflammatory Cytokine Levels Between Wild-Type HBV and Pre-core (G1896A) or Basal Core Promoter (A1762T/G1764A) Mutants

**DOI:** 10.3389/fmicb.2020.01653

**Published:** 2020-07-14

**Authors:** Keith C. K. Lau, Shivali S. Joshi, Douglas J. Mahoney, Andrew L. Mason, Guido van Marle, Carla Osiowy, Carla S. Coffin

**Affiliations:** ^1^Department of Microbiology, Immunology and Infectious Diseases, Cumming School of Medicine, University of Calgary, Calgary, AB, Canada; ^2^Calgary Liver Unit, Division of Gastroenterology and Hepatology, Department of Medicine, Cumming School of Medicine, University of Calgary, Calgary, AB, Canada; ^3^Department of Microbiology and Immunology, Faculty of Medicine and Dentistry, University of Alberta, Edmonton, AB, Canada; ^4^Viral Hepatitis and Bloodborne Pathogens, National Microbiology Laboratory, Winnipeg, MB, Canada

**Keywords:** viral hepatitis, genetic variants, APOBEC3 family, *in vitro* characterization, HBV precore/basal core promoter mutations

## Abstract

**Background:**

Chronic hepatitis B virus (HBV) infection is the leading cause of hepatocellular carcinoma (HCC) world-wide. HBV variants, particularly the G1896A pre-core (PC) and A1762T/G1764A basal core promoter (BCP) mutations, are established risk factors for cirrhosis and HCC, but the molecular biological basis is unclear. We hypothesized that these variants result in differential HBV replication, APOBEC3 family expression, and cytokine/chemokine expression.

**Methods:**

HepG2 cells were transfected with monomeric full-length containing wild-type, PC, or BCP HBV. Cells and supernatant were collected to analyze viral infection markers (i.e., HBsAg, HBeAg, HBV DNA, and RNA). Cellular APOBEC3 expression and activity was assessed by quantitative real-time (qRT)-PCR, immunoblot, differential DNA denaturation PCR, and sequencing. Cytokine/chemokines in the supernatant and in serum from 11 CHB carriers (4 non-cirrhotic; 7 cirrhotic and/or HCC) with predominantly wild-type, PC, or BCP variants were evaluated by Luminex.

**Results:**

HBeAg expression was reduced in PC and BCP variants, and higher supernatant HBV DNA and HBV RNA levels were found with A1762T/G1764A vs. G1896A mutant (*p* < 0.05). Increased APOBEC3G protein levels in wild-type vs. mutant were not associated with HBV covalently closed circular DNA G-to-A hypermutations. Differences in cytokine/chemokine expression in culture supernatants, especially IL-13 were observed amongst the variants analyzed. Noticeable increases of numerous cytokines/chemokines, including IL-4 and IL-8, were observed in *ex vivo* serum collected from CHB carriers with PC mutant.

**Conclusion:**

HBV sequence variation leads to differences in HBV protein production (HBeAg) and viral replication in addition to altered host innate antiviral restriction factor (APOBEC3) and cytokine/chemokine expression.

## Introduction

The hepatitis B virus (HBV) is a significant global pathogen with ∼257 million chronic HBV (CHB) carriers worldwide (WHO, 2017). CHB can lead to cirrhosis, liver failure, and hepatocellular carcinoma (HCC). HBV chronicity is due to an ineffective host immune response and persistence of the intranuclear HBV minichromosome, covalently closed circular DNA (cccDNA), which is poorly targeted by currently approved reverse transcriptase inhibitors (nucleos/tide analog) therapies (Jemal et al., 2011). Due to the error-prone method of viral replication, the HBV exists as quasi-species within the host (Gao et al., 2015). HBV-related oncogenesis is complex and is influenced by viral characteristics such as genetic variants particularly within the X/basal core promoter (BCP)/pre-core (PC) region and integration into the host chromosomes contributing to genomic instability and hepatocarcinogenesis. Through next-generation sequencing (NGS), our group and others have demonstrated the variability of HBV within CHB carriers either with or without end-stage liver disease (cirrhosis and cancer) (Yan et al., 2015; Lau et al., 2018; Wu et al., 2018). Moreover, we have shown HBV genome integration in both liver and lymphoid cells in individuals with hepatic and extrahepatic malignancy (Lau et al., 2019, 2020).

HBV X/BCP/PC mutations (i.e., G1896A pre-core and A1762T/G1764A double mutants) are strong predictors of HCC risk (Chen et al., 2007; Yang et al., 2008; Park et al., 2014; Wang et al., 2016; Yu et al., 2016; Rajoriya et al., 2017) and frequently reported in large epidemiological studies of HBV-related HCC (Gao et al., 2015). However, there is a limited understanding of the underlying molecular mechanisms and cellular pathogenesis of viral sequence heterogeneity leading to end-stage liver disease. The G1896A pre-core mutation introduces a premature stop codon in the precore/core HBV transcript resulting in abrogated HBeAg production (Carman et al., 1989). The A1762T/G1764 double mutation is located within the BCP region of the HBV genome which influences the expression of both the pre-core/core and the pregenomic (pg) RNA transcripts. As a result of the double mutation, pgRNA transcript synthesis is favored. HBeAg protein synthesis is reduced by ∼30–50% whereas pgRNA expression and subsequent HBV genome replication doubles (Buckwold et al., 1996; Tong and Revill, 2016). Both the G1896A and A1762T/G1764A mutations are associated with the “HBeAg-negative hepatitis” phase of CHB. These mutants are frequently found in CHB carriers who experience hepatic flares and liver inflammation with HBeAg-negative serology (Rajoriya et al., 2017; Yuen et al., 2018). Clinically, HBeAg-negative CHB carriers are found to have significantly lower levels of HBV DNA (Yuen et al., 2018). There is conflicting data from *in vitro* studies on the replicative capacity of G1896A or A1762T/G1764A HBV compared to wild-type HBV (Jammeh et al., 2008; Samal et al., 2015; Koumbi et al., 2016).

The innate antiviral restriction factors apolipoprotein B mRNA editing enzymes (APOBECs) serve to inhibit retroviruses, such as human immunodeficiency virus (HIV), due to their cytidine deaminase activity which results in G-to-A hypermutations. The human APOBEC3 family comprises of seven members (A, B, C, DE, F, G, and H) which vary in their localization, regulation, and substrate preferences (Green and Weitzman, 2019). In both HBV infected patients and experimental models, the APOBEC3 family of proteins have been demonstrated to induce hyper-editing and degradation of HBV cccDNA with potential therapeutic applications (Günther et al., 1997; Turelli et al., 2004; Suspène et al., 2005; Noguchi et al., 2007; Lucifora et al., 2014; Xia et al., 2016). Interestingly, G-to-A hypermutations within HBV genomes due to APOBEC3 activity are disproportionately found in HBeAg-negative CHB carriers (Noguchi et al., 2005; Beggel et al., 2013). In addition to the antiviral effects, APOBEC3 proteins are endogenous carcinogens as nucleic acid editing enzymes. Indeed, multiple cancers, including HCC, have been associated with APOBEC3 expression and mutational activity (Alexandrov et al., 2013; Roberts et al., 2013; Deng et al., 2014). Although many retroviruses encode specific viral products which function to counteract the effects of APOBEC3s such as the HIV vif proteins (Goila-Gaur and Strebel, 2008), a similar viral factor has not been identified in HBV infection. We evaluated whether APOBEC3 activity and expression is altered by X/BCP/PC variants as a mechanism that increased risk of end-stage liver disease (i.e, HCC and cirrhosis) in CHB carriers harboring these mutants.

In the current study, we evaluated *in vitro* the functional properties of two clinically important genetic variants of HBV associated with HCC, the G1896A and A1762T/G1764A mutations. We hypothesized that there are differences amongst viral variants with regards to HBV replicative capacity and host innate antiviral responses. This study was designed to compare *in vitro* viral replication in the wild-type, G1896A, and A1762T/G1764A HBV variants. In addition, we aimed to analyze the effect of these variants on host cell innate responses, namely the APOBEC3 family expression and subsequent antiviral hyper-editing. Lastly, we assessed markers of host innate immune response by measuring cytokine and chemokine levels both in *in vitro* transient transfection models and *in vivo* clinical CHB carriers.

## Materials and Methods

### Construction of Full-Length HBV Plasmids and Site-Directed Mutagenesis

Plasmid DNA containing a full genome copy of the HBV genome was constructed in-house. Briefly, HBV full genome was amplified from phenol-chloroform DNA isolated from a high viral load clinical sample (>5 × 10^8^ HBV copies/mL; genotype C) with high-fidelity Phusion DNA polymerase (New England Biolabs, Whitby, Canada). HBV FG P1 and P2 primers for amplification (Supplementary Table S1) were derived from Günther et al. (1995). PCR amplicons were purified from a 0.9% agarose gel followed by digestion with *HindIII* (New England Biolabs) and ligated with T4 DNA ligase into the cloning vector pUC19 which was similarly prepared. Ligations were transformed into TOP10 *E. coli* chemically competent cells (Invitrogen, Carlsbad, United States) and successful clones were identified and confirmed via PCR and sequencing, respectively, with standard M13 primers thus generating the pUC19-HBV.wt plasmid (Supplementary Table S1).

Generation of G1896A and A1762T/G1764A mutants were achieved using the QuikChange II site-directed mutagenesis kit (Agilent Technologies, Santa Clara, United States), as per manufacturer’s protocol using the pUC19-HBV.wt and mutagenic primers (Supplementary Table S1). Purified plasmids (pUC19-HBV.g1896a and pUC19-HBV.dmut) were isolated from selected clones and subsequently sequenced in-house to confirm the mutagenesis.

### Cell Culture and Transfection of Linearized HBV

HepG2 cells (Millipore Sigma, Burlington, United States) were cultured in Dulbecco’s modified Eagle’s medium supplemented with 10% fetal bovine serum and 1% pen/strep (Invitrogen) at 37°C and 5% CO_2_. Usage of cell lines originating from human sources for research purposes was approved by the University of Calgary Ethics Board (ID# REB20-0933). For transfection, cells were seeded into 6 well plates at 7.5 × 10^5^ cells/well and incubated overnight. Each well of HepG2 cells were transfected with 266 ng of linearized full-genome monomeric HBV (∼7.5 × 10^10^ copies) using Lipofectamine^TM^ 3000 (Invitrogen) as per manufacturer’s protocols. Supernatant and cells were collected at 0.25, 0.5, 1, 2, 3, 5, and 7 days after transfection for analysis of HBV markers and cellular mRNAs or proteins. Linearized HBV was prepared by digestion of pUC19-HBV plasmids with *BspQI* (New England Biolabs). The desired 3.2 kb HBV full-genome fragments were then purified from 0.9% agarose gels before use in transfections. All subsequent analyses included three independent transfections that were performed alongside 500 ng of pcDNA3.1-GFP as a marker of transfection success and efficiency was evaluated by fluorescent microscopy. Transfections, handling of biohazardous material, and genetic modifications of HBV were performed in accordance to biosecurity measures implemented by the University of Calgary and Government of Canada including dedicated use of biosafety cabinets or other equipment, personal protective equipment, restricted access to materials, and appropriate personnel training.

### Isolation and Quantification of Supernatant HBV DNA and RNA

HBV DNA in extracellular viral particles were isolated from 500 μL of transfected cell supernatant using polyethylene glycol as previously published by Pollicino et al. (2006). Subsequently isolated viral particles were then digested with DNase I (Quanta Biosciences, Beverly, United States) to remove any residual DNA from the transfection. Viral DNA was extracted from isolated particles using standard phenol-chloroform extraction and quantified using an in-house HBV DNA qPCR. In brief, the iTaq Universal SYBR Green (Bio-Rad Laboratories, Hercules, United States) was utilized alongside a plasmid standard for the purpose of HBV DNA quantification in supernatant. The qPCR primers used were designed to create an amplicon (i.e., HBV 1690–1917 nt) that spans the ends of the linearized HBV DNA to reduce the detection of residual transfected DNA (Supplementary Table S2).

Total RNA in the supernatant was isolated from 500 μL of transfected cell supernatant with TRIzol^TM^ (Invitrogen) as per manufacturers’ instructions and eluted into 20 μL of RNase-free water. Potential DNA contaminants were digested with DNase I (Quanta Biosciences) before specific amplification of HBV RNA using the Rapid Amplification of Complementary DNA (RACE) technique targeting HBV poly-A region (van Bommel et al., 2015). HBV RNA was subsequently quantified with the PerfeCTa FastMix II (Quanta Biosciences) and dilution of a plasmid standard.

Negative controls for qPCR include no template controls (water) and mock DNA/RNA extraction samples. Reverse transcriptase negative samples were also tested to confirm a lack of DNA contaminants in supernatant RNA experiments. All samples were performed in triplicate.

### Detection of Cellular HBV cccDNA

HBV cccDNA was isolated from transfected HepG2 cells by the Hirt extraction as previously described (Cai et al., 2013; Lau et al., 2018). Post-extraction T5 exonuclease digestion was performed prior to quantification of cellular cccDNA using qPCR (Lucifora et al., 2014; Xia et al., 2017). In brief, the iTaq Universal SYBR Green (Bio-Rad Laboratories) was utilized alongside an in-house plasmid standard containing the amplicon and specific primers encompassing the HBV nicked region (92F and 2251R, Supplementary Table S2) for cccDNA quantification. Due to high Ct values (> 35), the low level cellular cccDNA was detected using our previously published nested PCR technique targeting the nicked region of HBV (cccDNA direct and nested primers, Supplementary Table S2) (Lau et al., 2018). Approximately, 20–30 μg of Hirt extracted DNA prior to T5 digestion was utilized for nucleic acid hybridization (NAH) by first size separation on a 0.9% agarose gel followed by transfer to a nylon membrane (Amersham Hybond N+). The membrane was probed with subsequent wash and detection using the Roche DIG Wash and Block Buffer set and DIG Nucleic Acid Detection kit. Probes for NAH were created with the Roche PCR DIG probe synthesis kit targeting the X/BCP/preC, preC/Core, and surface genome regions (Supplementary Table S2). Equal proportions of these resulting probes were utilized for NAH. A NAH marker and positive control containing 3.2, 2.1, and 1.7 kb fragments of HBV was created from restriction enzyme digestion of plasmids containing a full genomic HBV dimer. Negative controls for NAH includes mock Hirt extraction.

### Analysis of Supernatant HBsAg, HBeAg, and Cytokines/Chemokines

Quantification of HBsAg in supernatant was done with an in-house sandwich ELISA. In brief, 100 ng of capture anti-HBsAg (Fitzgerald 10-H05H, clone M701077) was loaded into each well of protein binding plates (Corning, United States) in antibody binding buffer (Na_2_CO_3_/NaHCO_3_ with pH 9.6) and incubated overnight at 4°C. Plates were blocked with 2% w/v BSA in PBS for 1 h rocking at 37°C before addition of 100 μL of sample supernatant and incubated overnight at 4°C. Captured HBsAg was detected with 1:2000 of anti-HBsAg HRP-conjugate (Fitzgerald 60C-CR2100RX) in 2% BSA incubated for 1 h rocking at 37°C. Samples were then incubated with 100 μL of TMB (Thermo Fisher Scientific) followed by addition of an equal volume of HCl and measurement of sample absorbance at 450 nm. A standard curve of HBsAg protein (Fitzgerald 30C-CP2019R) diluted in HepG2 cell culture media was constructed for quantification of sample HBsAg. Positive signals were defined as 3 times the absorbance value of blank wells and all samples were performed in triplicates.

Presence of HBeAg within the supernatant was detected by the electrochemiluminescence EIA via the COBAS e411 platform (Elecsys; Roche Diagnostics, Laval, Canada). Cytokines and chemokines present in supernatant collected 12 h post-transfection was analyzed using a 42-plex Luminex discovery assay (Eve technologies, Calgary, Canada).

### Detection of Viral Proteins and APOBEC3G by Western Blot

Total protein was isolated from transfected cells with a SDS-based lysis buffer followed by protein concentration determination using the Bio-Rad DC^TM^ Protein assay as per the manufacturers’ protocol (Bio-Rad Laboratories). Thirty micrograms of protein was loaded onto a 12% Sodium Dodecyl Sulfate Polyacrylamide (SDS-PAGE) gel and the subsequent resolved gel was transferred to a nitrocellulose membrane (Amersham Protran 0.45 NC). Membranes were blocked with 5% skim milk before exposure to primary antibody: 1:1000 anti-GAPDH mouse monoclonal (Invitrogen 39-8600); 1:100 anti-HBsAg mouse monoclonal (Santa Cruz sc-53299); 1:500 anti-HBcAg mouse monoclonal (Abcam ab8637); 1:1000 anti-APOBEC3G rabbit polyclonal (Abclonal #A1459). Secondary antibody used was anti-mouse IgG HRP conjugated (GE healthcare NA-931) at 1:10000 (for GAPDH) and 1:1000 (for anti-HBsAg and anti-HBcAg) or anti-rabbit IgG HRP conjugated (GE healthcare NA-934) at 1:1000 (for APOBEC3G). Blots were developed with Immobilon^®^ Forte Western HRP substrate (Millipore Sigma). Densitometry analysis was performed using the Image Studio^TM^ Lite software version 5.2 (LI-COR Biosciences, Lincoln, United Kingdom).

### Semi-Quantitative Analysis of APOBEC mRNAs by qRT-PCR

Cellular mRNA was analyzed by isolating for total RNA with TRIzol^TM^ (Invitrogen) as per manufacturers’ instructions and eluted into 20 μL of RNase-free water. Potential DNA contaminants were removed with DNase I (Quanta Biosciences) before cDNA synthesis using the qScript cDNA supermix (Quanta Biosciences). APOBEC3 mRNA fold change was calculated by amplification and normalization alongside the internal reference, GAPDH, using the iTaq Universal SYBR Green (Bio-Rad Laboratories, primer sequences can be found in Supplementary Table S3). Negative controls for quantitative real-time (qRT)-PCR include no template controls (water), reverse transcriptase negative, and mock RNA extraction samples. All samples were performed in triplicate.

### Evaluation of APOBEC3 Hypermutation Activity

3D-PCR was performed on the qPCR-amplified cccDNA products to identify the presence of HBV genome hyper-editing by APOBEC proteins based on the methodology of Xia et al. (2017). In brief, HBx in primers (HBV 1374–1586 nt) were utilized as internal primers for the qPCR products within a range of denaturation temperatures (95–82°C). Positive bands visualized on a 1.3% agarose gel were excised and sequenced in-house (University of Calgary Core DNA facility, Calgary, Canada). To further evaluate for APOBEC3 hypermutation activity, excised amplicons from the cccDNA nested PCR used for detection was inserted into the pGEM-T easy vector and transformed into TOP10 *E. coli* for clonal sequencing.

### CHB Patient Sample Collection and Luminex Analysis

11 CHB carriers (4 non-cirrhotic, 1 cirrhosis and 6 with cirrhosis and HCC), enrolled from our previously published study (Lau et al., 2019, 2020) from the University of Calgary Liver Unit were recruited. This study was approved by the U of C conjoint health research ethics board, CHREB (Ethics ID# REB15-3137). Whole blood was collected, from which serum was isolated for analysis in the 42-plex Luminex discovery assay (Eve technologies). Published NGS sequencing data (Lau et al., 2019, 2020) showed the proportion of A1762T, G1764A, and G1896A mutants in the HBV quasi-species population allowed grouping of CHB carriers into three cohorts: predominantly wild-type (<50% of any mutant; *n* = 4), G1896A (>50% of only G1896A; *n* = 3), or A1762T/G1764A (>50% of only A1762T and G1764A; *n* = 4) (Supplementary Table S4). Clinical data, laboratory assays, and HBV tests including HBV DNA, quantitative HBsAg, HBeAg, and anti-HBe was determined with clinical PCR (Abbott or Roche TaqMan PCR) and commercial chemiluminescent microparticle immunoassays (Abbott Architect).

### Statistical Analysis

Quantifiable supernatant HBV markers (i.e., HBsAg, HBV DNA, and HBV RNA) were compared using linear regression models. Comparison of densitometry relative expression, qPCR fold changes, and cytokine/chemokine concentrations were performed using one-way ANOVA with the *post-hoc* Bonferroni’s multiple comparisons test. All statistical analysis testing used the GraphPad Prism version 6.0 with a level of significance of 0.05.

## Results

### Viral Replication Markers, With the Exception of HBeAg, Are Comparable Between X/BCP/PC Mutants and Wild-Type HBV in Cultured Liver Cells

**FIGURE 1:**
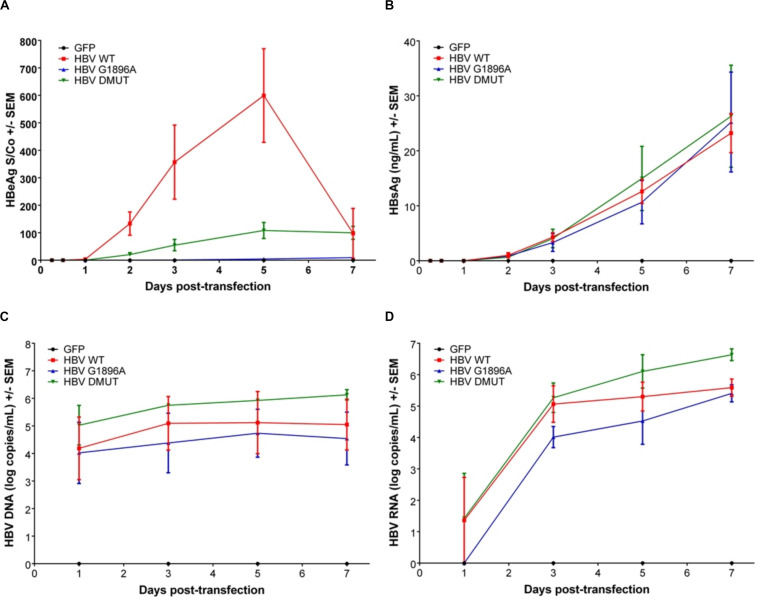
Differences in secreted HBV infection markers in HepG2 cell supernatant after transfection with wild-type vs. PC or BCP mutant HBV genomes. HepG2 cells were co-transfected with pcDNA-GFP +/- linearized wild-type (WT), G1896A or A1762T/G1764A (DMUT) HBV. Cell supernatant was analyzed post-transfection for **(A)** HBeAg; **(B)** HBsAg; **(C)** HBV DNA; and **(D)** HBV RNA. HBeAg was decreased in mutant compared to wild-type and differences in HBV DNA (*p* = 0.0363) and HBV RNA (*p* = 0.0220) were noted between G1896A vs. A1762T/G1764A mutants. Compiled results of three independent transfections.

Viral replicative markers including HBsAg, HBV DNA, HBV RNA, and HBeAg in the cell supernatant were compared amongst each variant (Figures 1A–D). As expected, HBeAg expression differed with the greatest levels in the wild-type HBV and clearly reduced levels in the A1762T/G1764A double mutant (Figure 1A). Secretion of HBsAg is comparable between the tested HBV mutants and wild-type HBV. In addition, HBV DNA isolated from the supernatant was quantified, with only minimal increases over the course of the transfection experiments (Figure 1C). Secreted HBV RNA species is a relatively novel clinical biomarker that have yet to be explored between HBV genetic variants *in vitro*. Here, we demonstrate that no significant differences are present between the X/BCP/PC mutations and the wild-type (Figure 1D). Interestingly, the A1762T/G1764A double mutation HBV has consistently higher levels of supernatant HBV DNA (*p*-value = 0.0363) and HBV RNA (*p*-value = 0.0220) in comparison to G1896A HBV variant.

**FIGURE 2:**
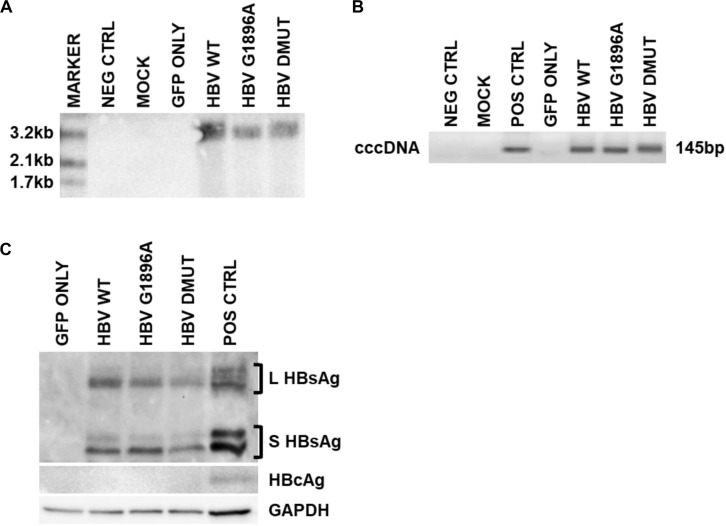
Detection of intracellular HBV replication and infection markers following transfection with wild-type vs. mutant HBV genomes. Representative images of 3 days post-transfection cellular cccDNA extracts show undetectable cccDNA by **(A)**. Direct nucleic acid hybridization (without T5 exonuclease digestion), but identification with the more sensitive **(B)** nested PCR (with T5 exonuclease digestion) targeting the HBV nicked region. PCR positive control used was a full-length HBV plasmid. In addition, **(C)** viral surface protein species were detected within the cellular lysates via immunoblot, but not HBV core proteins likely due to low levels. Immunoblot positive control used was liver tissue protein extracts from a chronically infected HBV individual. Representative results of three independent transfections.

Cellular HBV replication and protein markers were also assessed as well as transfection efficiency (Supplementary Figure S1). In all cases and timepoints, HBV cccDNA was unquantifiable by qPCR, due to low level production. Similarly, direct NAH of Hirt extracts without T5 digestion only identified other forms of HBV DNA species (i.e., relaxed circular or double stranded linear DNA) (Figure 2A). Thus, in order to detect and compare HBV cccDNA, a nested PCR targeting the HBV nicked region with different primers than the qPCR effectively identified viral cccDNA samples extracted by Hirt with T5 exonuclease digestion (Figure 2B). Similar levels of cccDNA were observed between the different variants of HBV. Western blot analysis was used to identify intracellular viral protein including HBsAg species and HBcAg (Figure 2C). However, HBcAg was also undetectable, likely due to low level expression within the transfected cells.

### Differences in APOBEC3 Family Expression Between Wild-Type vs. Mutant HBV Do Not Result in Alterations in HBV cccDNA Hypermutation in Hepatoma Cells

**FIGURE 3:**
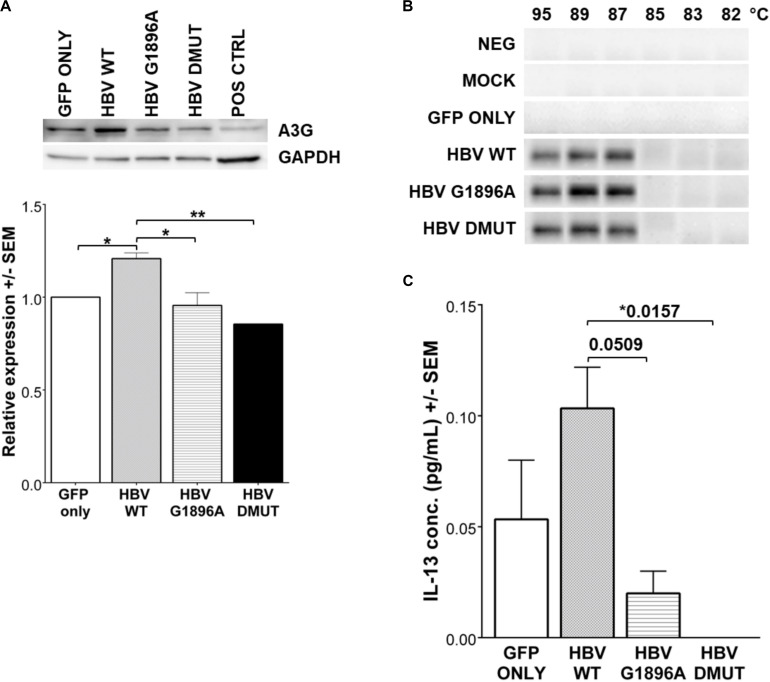
Expression of IL-13 and APOBEC3G (A3G) is reduced in HBV X/BCP/PC mutants, but do not result in any mutational changes in HBV cccDNA. **(A)** A3G protein was detected by western blot with densitometry analysis 1-day post-transfection. Reduced A3G protein expression was found in mutants vs. WT. Positive control was liver explant tissue from chronically infected HBV carrier. **(B)** 3D-PCR was used to assess for APOBEC3 family hypermutation activity of HBV genomes at 1-day post-transfection. **(C)** Cell supernatant was collected at 12 h reveals that secreted immune factor IL-13 had differential expression patterns amongst the wild-type and mutant HBV. Relative expression and cytokine concentrations were analyzed using one-way ANOVA with *post-hoc* Bonferroni’s multiple comparisons test: *0.01 < *p*-value < 0.05; **0.001 < *p*-value < 0.01. Compiled results of three independent transfections.

APOBEC3 family gene expression was evaluated by qRT-PCR in HepG2 cells transfected with linearized HBV DNA. The presence of the viral DNA or different variants did not result in significant changes in APOBEC3 family mRNA induction (*p*-values > 0.5) (Supplementary Figure S2A). Further, an evaluation of APOBEC3G protein levels by immunoblot showed increased amounts in wild type compared to the mock and HBV X/BCP/PC variants (Figure 3A) which was confirmed with densitometry analysis (*p*-values: vs. mock = 0.0414; vs. G1896A = 0.0205; vs. A1762T/G1764A = 0.0058). Subsequent analysis of APOBEC3 activity between HBV variants was assessed using 3D-PCR. Prior studies have found that HBV DNA species show G-to-A hypermutations via the APOBEC3 family members (Günther et al., 1997; Turelli et al., 2004; Suspène et al., 2005; Noguchi et al., 2007; Lucifora et al., 2014; Xia et al., 2016), however, no visible differences in DNA denaturation was observed in the cccDNA 1 day post-transfection between the X/BCP/PC variants and wild-type HBV (Figure 3B). Indeed, sequencing data analysis showed similar levels of nucleotides (particularly G and A bases) present even amongst PCR products from lower denaturation temperatures (Supplementary Figure S2B). This lack of hypermutation of the viral cccDNA was observed even at later time points including 3, 5, and 7 days post-transfection (Supplementary Figure S3). To confirm the results of the 3D-PCR hypermutation assays, clonal sequencing of the cccDNA nested PCR products was performed and revealed a lack of HBV cccDNA G-to-A hypermutation (Supplementary Figure S4).

### Expression of Cytokines/Chemokines Differs Amongst HBV Variants

Cytokines and chemokines within the supernatant were evaluated 12 h post-transfection to identify differences in hepatocyte innate immune responses upon exposure to HBV. Analysis of cytokines/chemokines revealed differences in Interleukin (IL)-13 between the wild-type and variants tested. It is interesting to note that both of the X/BCP/PC variants lead to suppression of this cytokine in comparison to wild-type (*p*-values: vs. G1896A = 0.0509; vs. A1762T/G1764A = 0.0157; Figure 3C). Other cytokines/chemokines analyzed including IL-12P40, MDC, TGF-α, and sCD40L did not have significant differences in expression levels amongst the wild-type and variant HBV (Supplementary Figure S5). In addition, serum derived from both HBeAg positive and negative CHB carriers containing these mutations were analyzed (Supplementary Table S4). Similarly, the analyzed cytokines/chemokines were comparable in the individuals with predominantly G1896A variant (Supplementary Figures S6, S7).

## Discussion

HBV genetic variants have been identified with clinical implications including antiviral treatment response, immune escape, and increased risk to HCC (Gao et al., 2015). Prior epidemiological and population-based studies have demonstrated the associations of X/BCP/PC variants, especially the G1896A pre-core and the A1762T/G1764A double mutation to cirrhosis and HCC (Chen et al., 2007; Yang et al., 2008; Park et al., 2014; Wang et al., 2016; Yu et al., 2016; Rajoriya et al., 2017). However, there is a limited understanding of the molecular mechanisms and pathogenesis underlying this association. There are few studies on HBV genetic variants and their functional impact on the novel viral biomarkers (i.e., serum RNA), APOBEC3 family, and host cytokine/chemokine responses. The current study evaluates these clinically relevant mutations using an *in vitro* transient transfection model in parallel with representative clinical samples with either wild-type, PC, or BCP mutants. Viral replication and infectious markers were assessed and clear differences between the X/BCP/PC variants were demonstrated. In addition, this model was used to evaluate whether these variants would induce APOBEC3 expression and activity and hence provide a plausible biological mechanism for carcinogenesis risk. Although we found reduced APOBEC3G protein expression in X/BCP/PC mutants compared to wild-type HBV, it did not impact APOBEC3 cytidine deaminase activity on the viral cccDNA.

In the current study, HBeAg expression was reduced and we found noticeable differences in the replication of G1896A and A1762T/G1764A mutations *in vitro*. The double mutation variant of HBV had more robust HBV replication, evident through the higher levels of supernatant HBV DNA and RNA. We also found increased HBV DNA levels and detection within the A1762T/G1764A mutant compared to the G1896A mutant in agreement with these *in vitro* results. However, it is important to note that our detection of HBV DNA does not confirm the presence of infectious virions. Although our methodology included PEG precipitation and DNase digestion to selectively isolate encapsidated HBV DNA, the detection of contaminating HBV DNA potentially from remnant transfection DNA or dead cells that escaped nuclease degradation remains a possibility. Serum HBV RNA is a promising biomarker for cccDNA activity and expression that may have clinical utility for disease monitoring or predictor of treatment outcomes (Liu et al., 2019). Indeed, a report by van Bommel et al. (2015) demonstrated the association of serum HBV RNA to HBeAg seroconversion in patients treated with nucleos/tide analog therapies. More recently, a study by van Campenhout et al. (2018) showed the association of serum RNA to established markers of HBV and liver inflammation including HBV DNA, HBeAg, and alanine transaminase (ALT). Interestingly, the investigators also reported that the presence of BCP mutations (i.e., A1762T/G1764A) correlated to lower levels of serum HBV RNA. However, these *in vivo* findings are in contrast with our *in vitro* results in which secreted HBV RNA from the A1762T/G1764A double mutant were not significantly reduced in comparison to the wild-type HBV. These differences might be due to the limitations of the transient transfection model used in our study and the inability to quantify cccDNA levels for correlation with HBV RNA secretion. Indeed, our challenges in quantification and detection by Southern blot of cccDNA is a notable limitation. In our study, we were only able to identify HBV cccDNA using highly sensitive nested PCR techniques which may amplify other contaminant forms of HBV genetic material that escaped T5 exonuclease digestion.

We used a well-established system with transfection of genotype C HBV into HepG2 cells. However, there are conflicting studies on the *in vitro* replicative capacity of the X/BCP/PC mutants which could arise from differences amongst the cell lines (i.e., Huh 7 and HepG2) used for the transfection by other investigators (Buckwold et al., 1996; Moriyama et al., 1996; Scaglioni et al., 1997; Jammeh et al., 2008; Samal et al., 2015; Koumbi et al., 2016). It is also possible that the HBV DNA construct used for transfection (either a multimeric or monomeric genome) may impact *in vitro* findings as suggested by Samal et al. (2015). Further, differences between the results of *in vitro* models may arise from other mutations, variants, and/or genotypes of the HBV DNA. Indeed, independent studies by Cui et al. (2015) and Sozzi et al. (2016) presents genotypic differences amongst viral replication using Huh7 and HepG2 transfection models.

The APOBEC3 family members are increasingly recognized as carcinogenic when overexpressed and are frequently dysregulated or show mutations in cancer (Alexandrov et al., 2013; Roberts et al., 2013). Further, thee association of the APOBEC3 family as innate restriction factors to retrovirus and HBV infection is well established (Turelli et al., 2004; Suspène et al., 2005; Okada and Iwatani, 2016). Thus, in our current study we assessed whether X/BCP/PC mutants and reduced HBeAg levels are linked to increased HCC risks via APOBEC3 overexpression and excessive activity. Despite the potential differences in APOBEC3G expression identified by immunoblot, no clear differences in hyper-editing activity of the cccDNA between HBV variants were found by 3D-PCR. However, research largely from retrovirus (i.e., HIV) studies demonstrates that APOBEC3G is typically incorporated into new virions and the anti-viral effects are primarily noticeable in subsequently infected cells (Desimmie et al., 2014). The current transient transfection model of HepG2 does not allow subsequent HBV infection and transmission. Further, it is important to consider that APOBEC3 anti-viral activity might be more extensive than viral genome hyper-editing, particularly in HBV infection (Janahi and McGarvey, 2013). Indeed, APOBEC3G with defective cytidine deaminase activity retains anti-HBV effects via inhibition of reverse transcription (Nguyen et al., 2007).

The strongest inducers of APOBEC3 expression are interferons (IFN) (Covino et al., 2018). In the current study, the levels of IFNα and IFNγ in cell supernatant may have been insufficient to induce APOBEC3 expression. Our findings demonstrate that cytokine/chemokine expression, with the exception of IL-13, do not significantly differ between the HBV wild-type vs. X/BCP/PC variants. To enhance the clinical relevance of the *in vitro* cytokine analysis, we evaluated serum derived from CHB patients with predominantly wild-type, G1896A, and A1762T/G1764A. Similar to the *in vitro* findings, CHB carriers with different HBV variants had comparable cytokine and chemokine levels. It is intriguing to note that a consistent elevation of numerous immune markers was observed, as well as elevated ALT, within the G1896A variant which might suggest overall heightened immune activity and hepatic inflammation within these individuals. However, the small sample sizes and the large variability in cytokine/chemokine expression levels amongst the clinical samples prevents firm conclusions to be made and warrants additional larger scale studies.

Due to the overlapping nature of the HBV genomes, the X/BCP/PC mutants evaluated would also induce mutations within HBx which has known carcinogenic effects. The A1762T/G1764A mutations are responsible for the K130M/V131I mutations found in the HBx. Recent independent reports by Siddiqui et al. (2017) and Chiu et al. (2019) linked mutant HBx to hepatocarcinogenesis, however, these investigators studied an overexpression model using an HBx construct without HBV infection and viral proteins/DNA. An intriguing connection of HBx to APOBEC3B was identified by Shen et al. (2020) and demonstrated the up-regulation of DHX9 proteins by HBx. In their related study, Chen et al. (2020) reported that DHX9 suppresses APOBEC3B and inhibited the anti-HBV effects. Additionally, HBx post-translational suppression of APOBEC3G has been shown within Huh7 cells (Chen et al., 2017). Overall, the pleiotropic effects of HBx may underly the impact of HBV genetic variants and APOBEC3 family activity.

The current study provides novel data on HBV genetic variants and their impact on viral fitness, potential association with innate antiviral restriction factors (i.e., APOBEC3 family), and host inflammatory cytokine/chemokine expression within a hepatocyte cell model system and in clinical samples from CHB carriers. HBV sequence variation impacts HBeAg expression, viral replication, APOBEC3 expression, and host immune response *in vitro*, and is supported by the analysis of clinical samples from CHB carriers with wild-type vs. mutant HBV infection. The results of this study enhance understanding of HBV sequence variations and their impact on host-cell interactions, host innate antiviral restriction factors and the pathogenesis of HBV-related HCC.

## Data Availability Statement

The raw data supporting the conclusions of this article will be made available by the authors, without undue reservation, to any qualified researcher.

## Ethics Statement

The studies involving human participants were reviewed and approved by the University of Calgary Conjoint Ethics Review Board. The patients/participants provided their written informed consent to participate in this study.

## Author Contributions

KL, AM, DM, GM, and CC contributed to the study conception and design. KL and CO acquired the data. KL, SJ, and CC were responsible for the analysis and interpretation of the data. KL and CC drafted and revised the manuscript. All authors approved the final version for journal submission.

## Conflict of Interest

The authors declare that the research was conducted in the absence of any commercial or financial relationships that could be construed as a potential conflict of interest.
