# Supplementary material for: Differences in HBV Replication, APOBEC3 Family Expression, and Inflammatory Cytokine Levels Between Wild-Type HBV and Pre-core (G1896A) or Basal Core Promoter (A1762T/G1764A) Mutants
Source: Front Microbiol. 2020 Jul 14;11:1653. doi: 10.3389/fmicb.2020.01653 (PMC7372132; doi:10.3389/fmicb.2020.01653)
Supplement: Supplementary file 1 [file Data_Sheet_1.PDF]

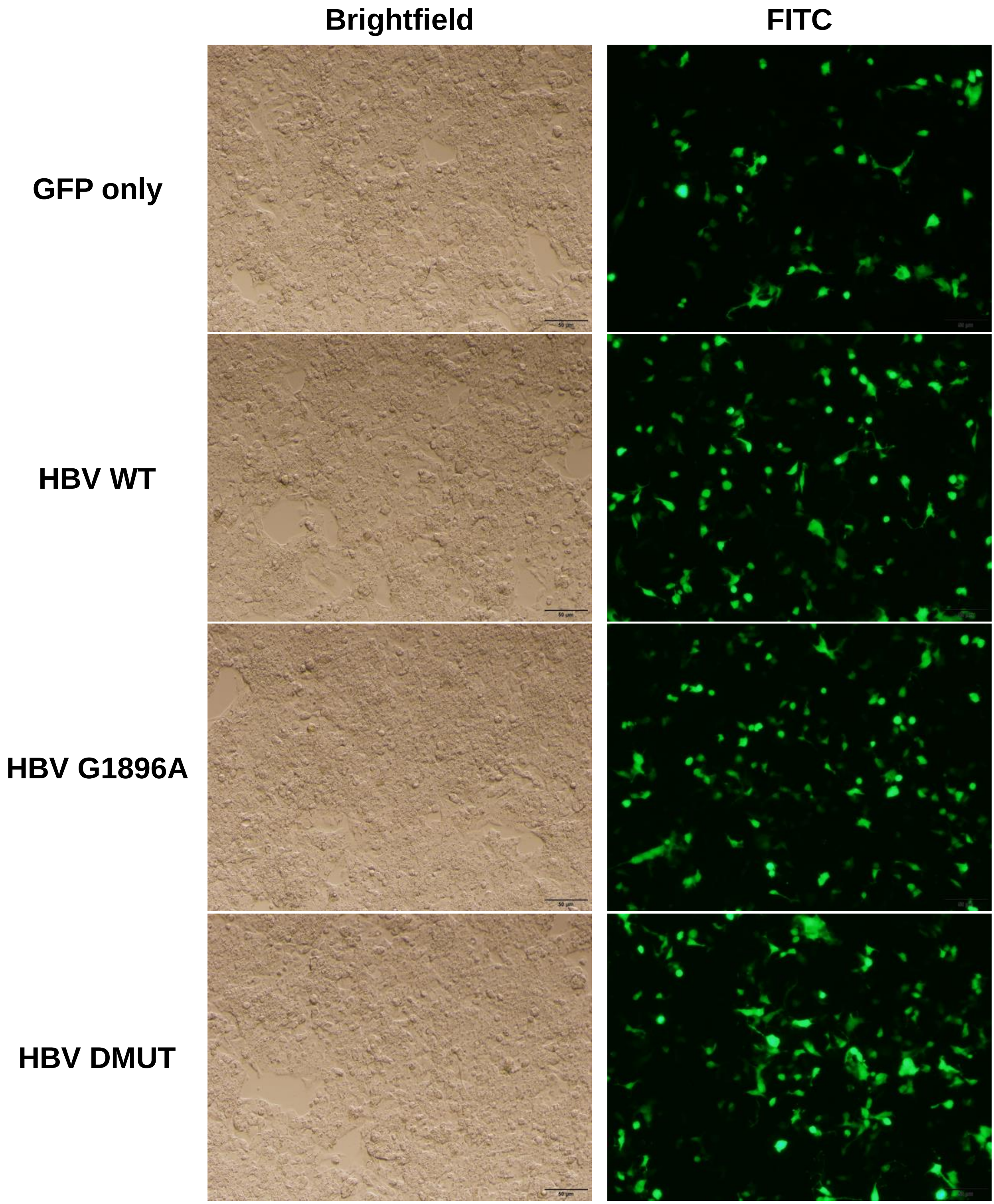

Figure S1. Representative images of transfection efficiency 3 days post-transfection using fluorescent microscopy.

Using both brightfield and FITC filters, cellular confluency and transfection efficiency was compared amongst the transfection conditions. WT = wild-type; DMUT = double mutant (A1762T/G1764A). Total magnification of 100X. Representative images of three independent transfections.

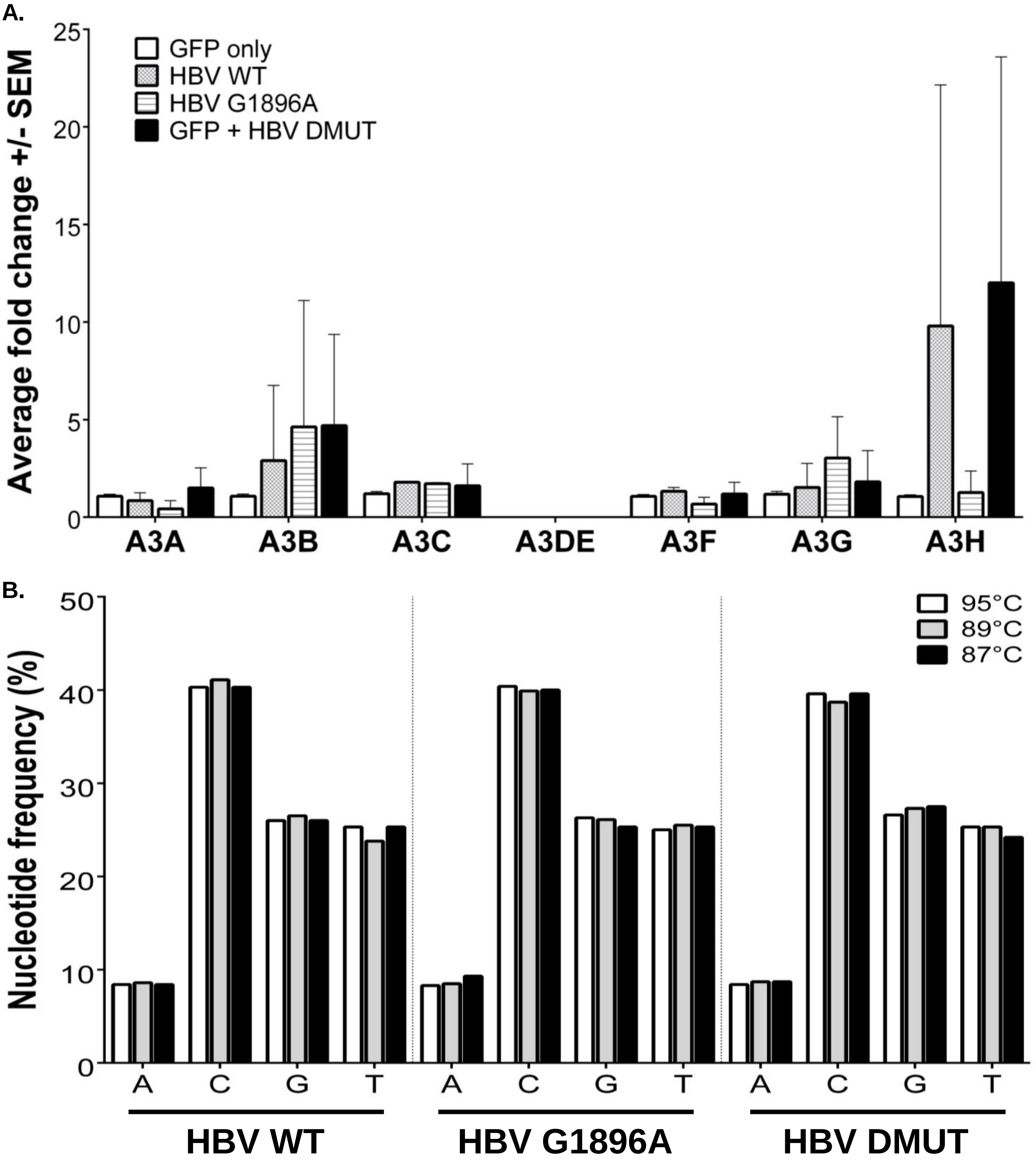

Figure S2. Reduced APOBEC3 (A3) cellular expression in HBV mutants and no HBV cccDNA hypermutation

Cellular A3 mRNA levels were evaluated 1-day post-transfection using GAPDH as an internal reference gene. **A.** Virally transfected DNA resulted in minimal non-significant induction of APOBEC3B, 3C, 3G, and 3H; or relative decreases of APOBEC3A and 3F. Average fold changes were analyzed by one-way ANOVA with post-hoc Bonferroni's multiple comparison test. **B.** 3D-PCR products 1-day post-transfection were sequenced to assess for APOBEC3 family activity with no significant changes in HBV cccDNA.

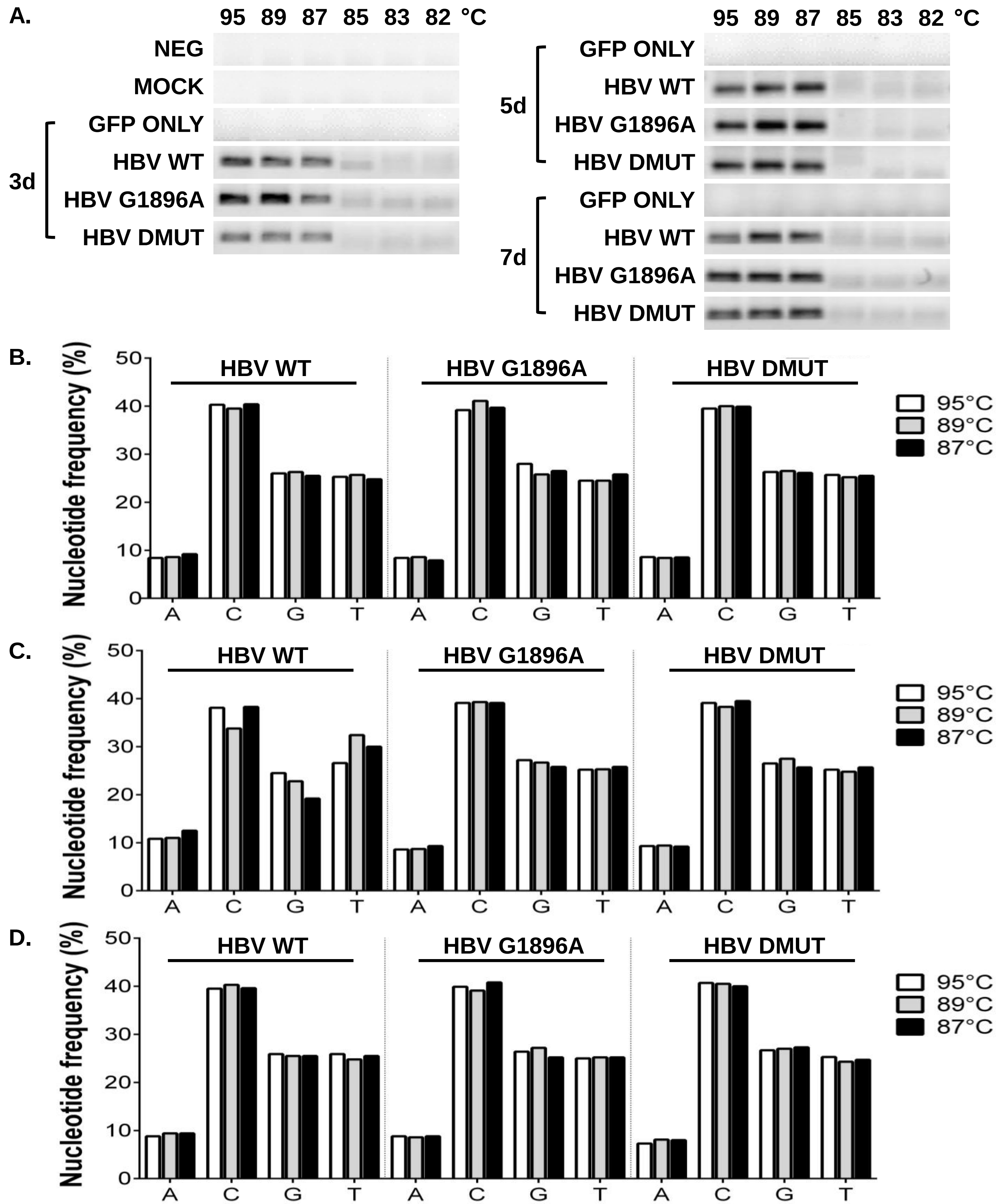

Figure S3. Lack of APOBEC3 hypermutation activity on HBV cccDNA even after 3-7 days post-transfection.

Hypermutation of HBV genomes were evaluated 3, 5, and 7 days post-transfection to assess for APOBEC3 family activity by **A.** 3D-PCR, and subsequent sequencing of the excised bands to identify nucleotide frequencies from wild-type (WT), G1896A, and A1762T/G1764A (DMUT) at **B.** 3 days post-transfection; **C.** 5 days post-transfection; and **D.** 7 days post-transfection.

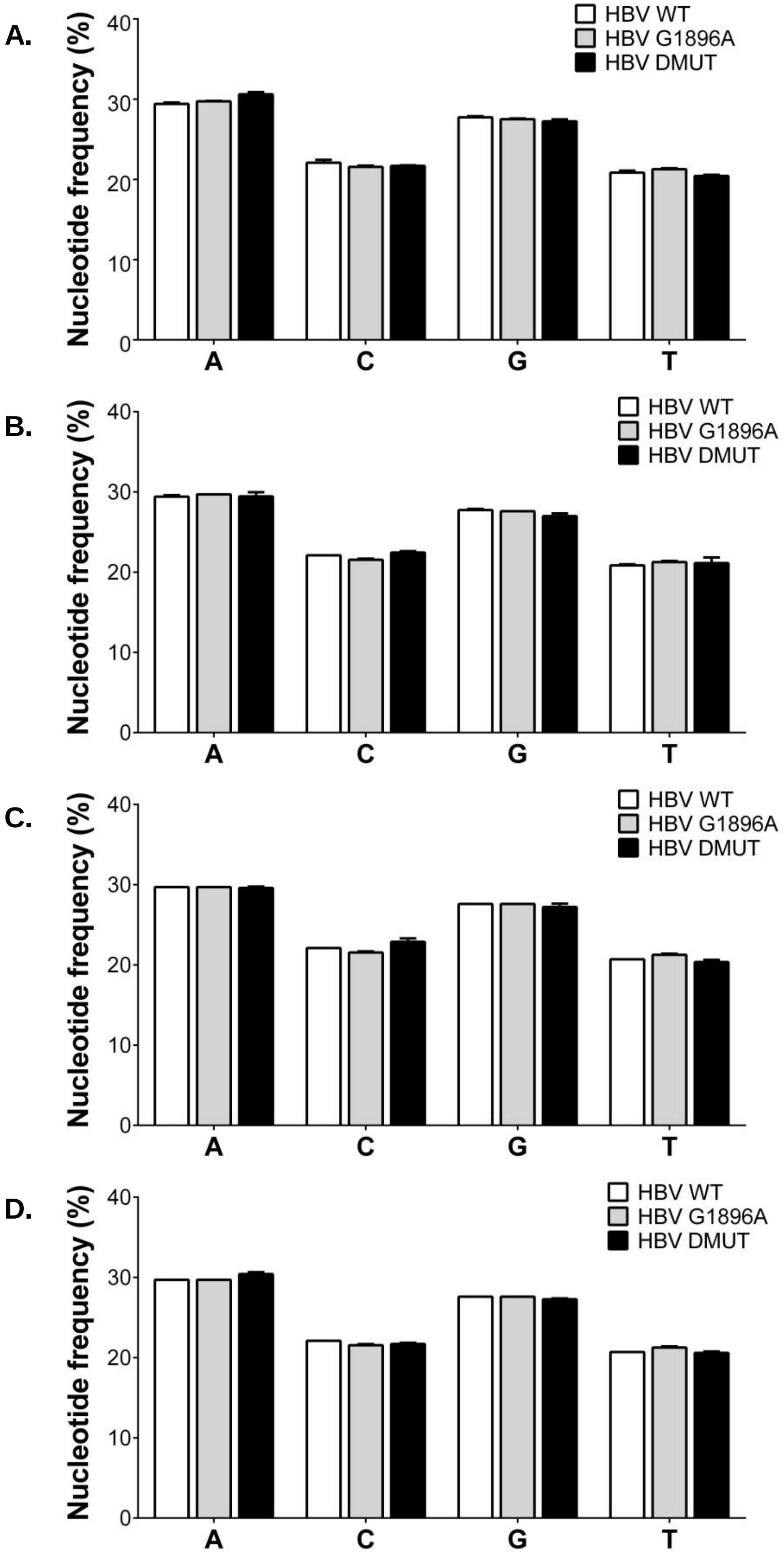

Figure S4. Evaluation of APOBEC3 hypermutation of HBV cccDNA by clonal sequencing. Nested cccDNA PCR products was used clonal sequencing by insertion of gel excised products into the pGEM-T easy vector. Frequency of each nucleotide from sequencing was plotted from the **A.** 1 day; **B.** 3 days; **C.** 5 days; and **D.** 7 days post-transfection. WT = wild-type.

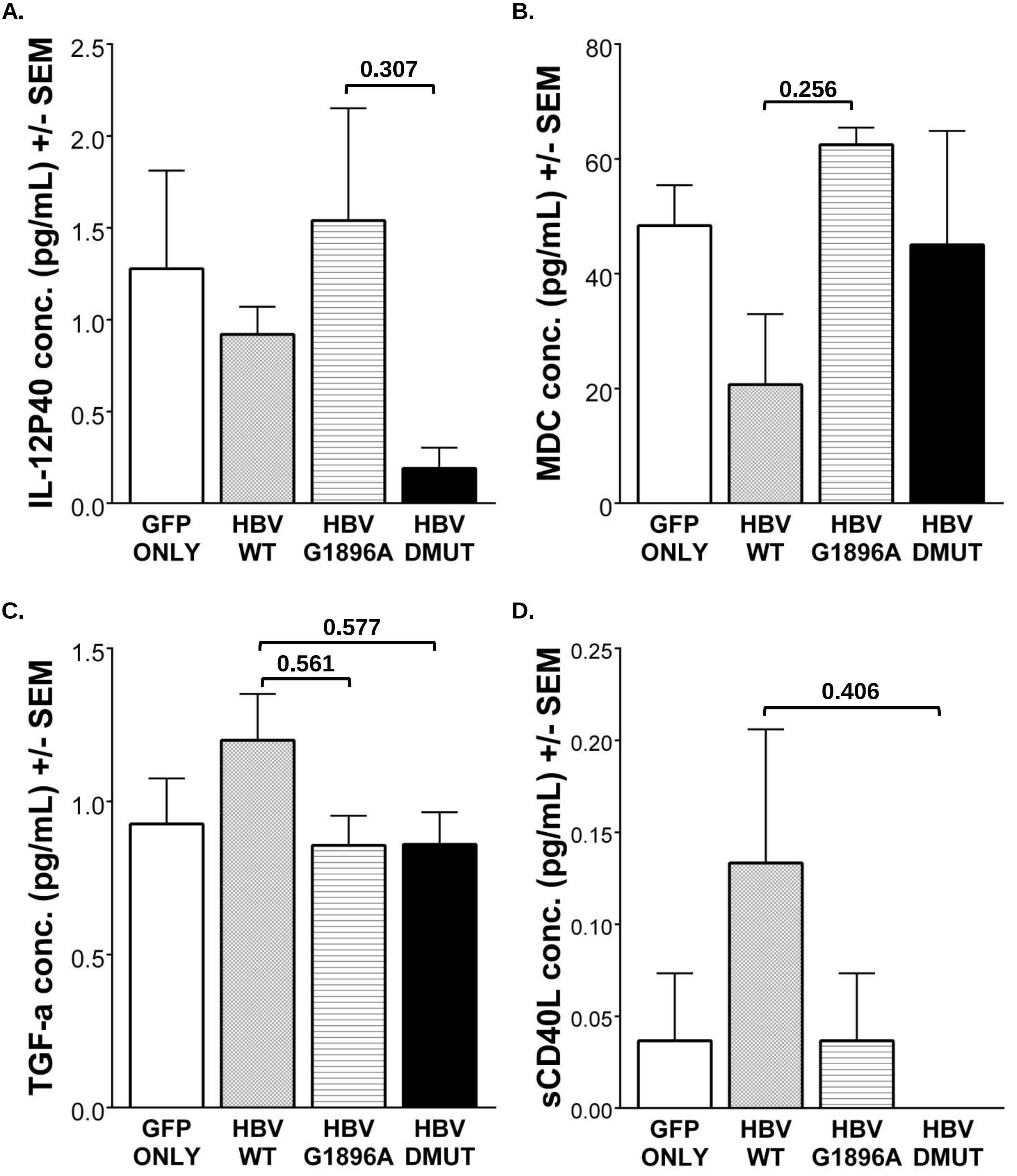

Figure S5. Cytokine/chemokine expression differs when transfected with different HBV variants. Supernatant collected 12 hours post-transfection was quantified for cytokines/chemokines by Luminex. The HBV variants resulted in different patterns of immune response induction as observed in many cytokines including: **A.** IL-12P40; **B.** MDC; **C.** TGF- α; and **D.** sCD40L. One-way ANOVA with post-hoc Bonferroni's multiple comparisons test were used for statistical analysis and numbers represent p-values.

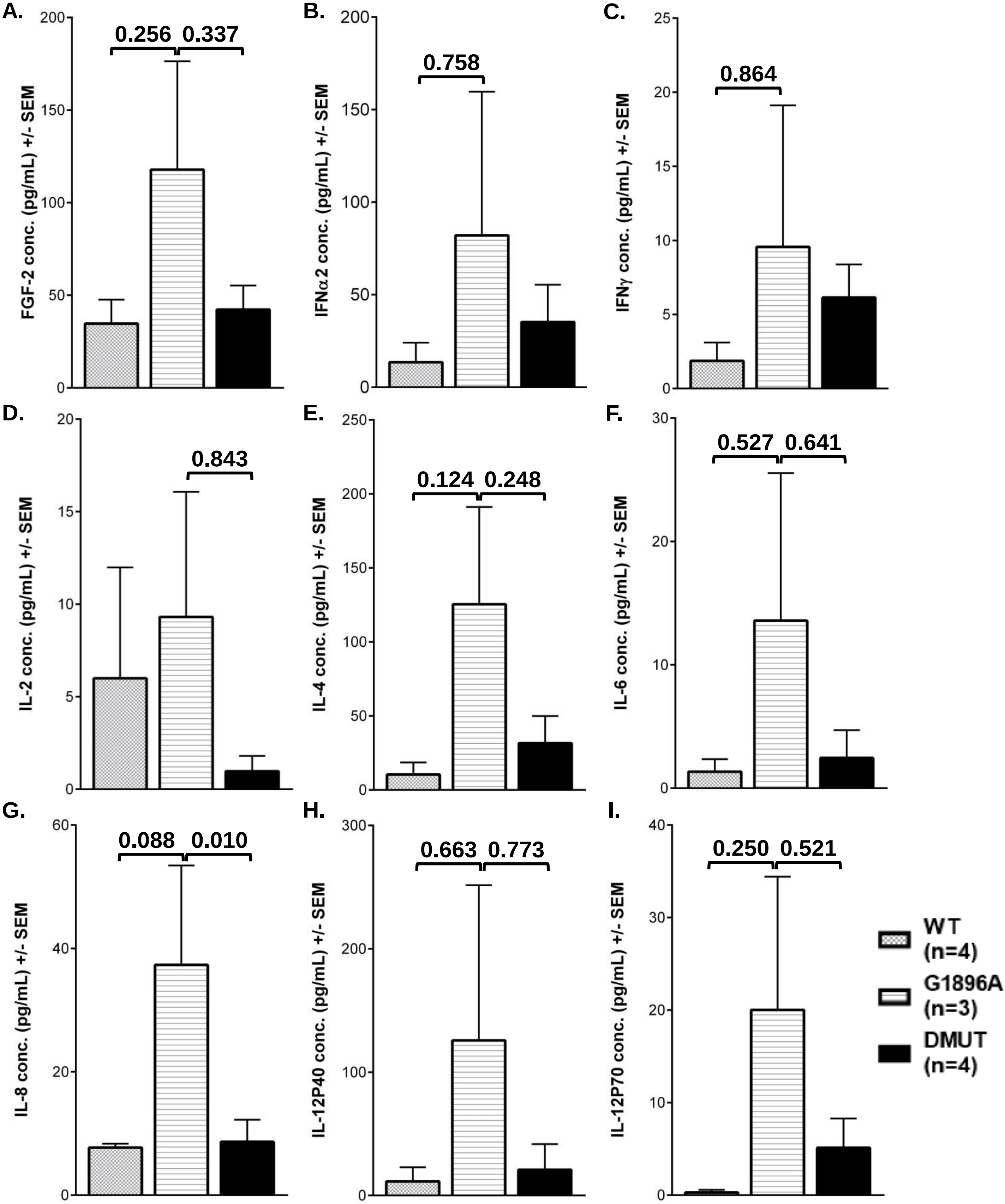

Figure S6. Elevation of cytokine/chemokine in CHB carriers with G1896A variant HBV. CHB carrier serum was quantified for cytokines/chemokines by Luminex. A general trend of elevated cytokines were observed in CHB carriers with G1896A variant including: **A.** FGF-2; **B.** IFNα2; **C.** IFNγ; **D.** IL-2; **E.** IL-4; **F.** IL-6; **G.** IL-8; **H.** IL-12P40; **I.** IL-12P70. One-way ANOVA with post-hoc Bonferroni's multiple comparisons test were used for statistical analysis and numbers represent p-values.

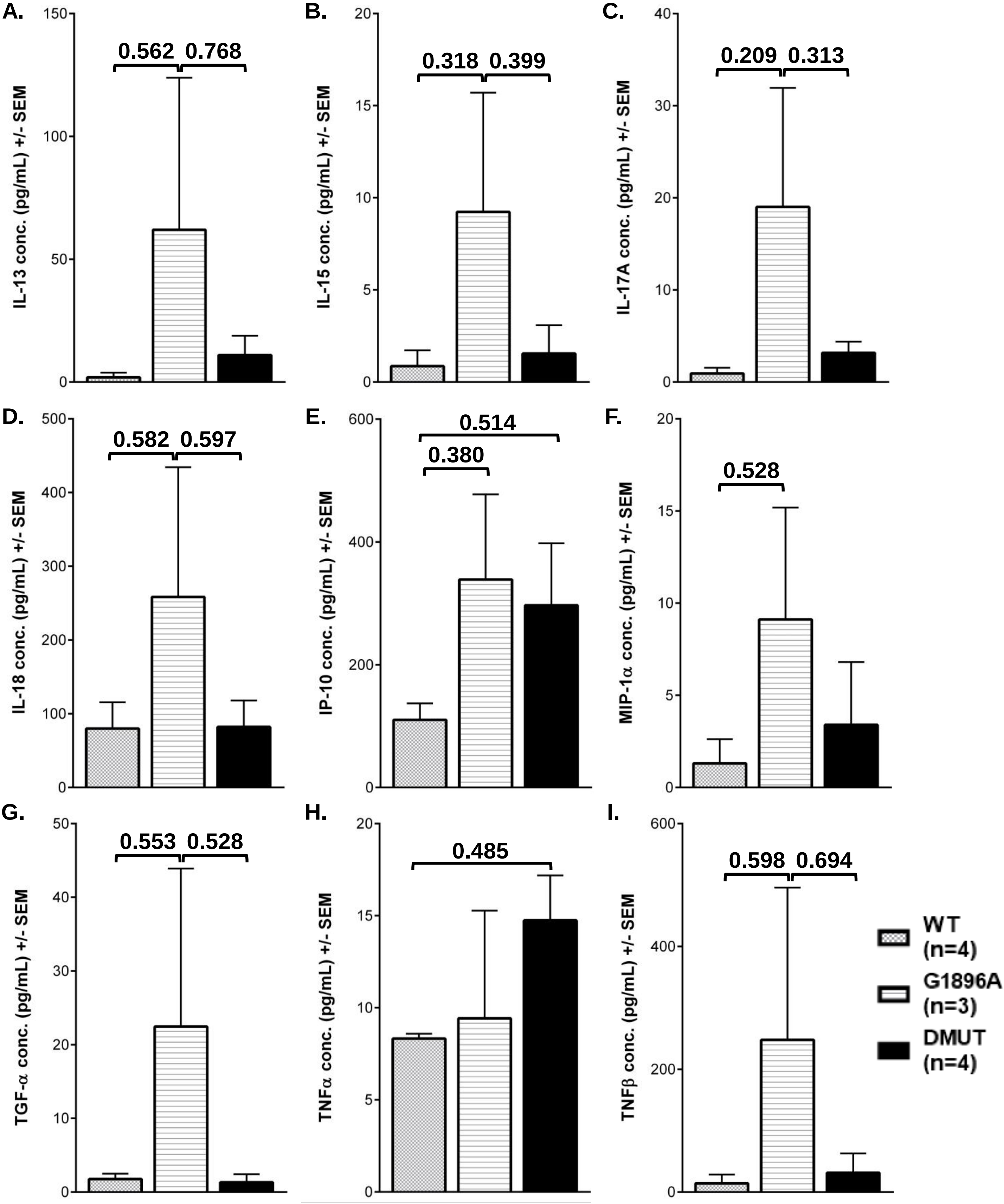

Figure S7. Elevation of cytokine/chemokine in CHB carriers with G1896A variant HBV.
CHB carrier serum was quantified for cytokines/chemokines by Luminex. A general trend of elevated cytokines were observed in CHB carriers with G1896A variant including: **A.** IL-13; **B.** IL-15; **C.** IL-17A; **D.** IL-18; **E.** IP-10; **F.** MIP-1α; **G.** TGF-α; **H.** TNFα; and **I.** TNFβ. One-way ANOVA with post-hoc Bonferroni's multiple comparisons test were used for statistical analysis and numbers represent p-values.
