# Supplementary material for: Differences in HBV Replication, APOBEC3 Family Expression, and Inflammatory Cytokine Levels Between Wild-Type HBV and Pre-core (G1896A) or Basal Core Promoter (A1762T/G1764A) Mutants
Source: Front Microbiol. 2020 Jul 14;11:1653. doi: 10.3389/fmicb.2020.01653 (PMC7372132; doi:10.3389/fmicb.2020.01653)
Supplement: Supplementary file 2 [file Table_1.DOCX]

**Table S1.** Primers used for full genome amplification and site-directed mutagenesis of the HBV wild-type genome. Underlined nucleotides represent the recognition site of HindIII used for cloning and mutant nucleotides are enlarged and bolded.

| **PRIMER** | **SEQUENCE (5’ TO 3’)** |
| --- | --- |
| **HBV FG P1** | CCGGAAAGCTTGAGCTCTTCTTTTTCACCTCTGCCTAATCA |
| **HBV FG P2** | CCGGAAAGCTTGAGCTCTTCAAAAAGTTGCATGGTGCTGG |
| **M13 FOR** | GTAAAACGACGGCCAGT |
| **M13 R-17** | CAGGAAACAGCTATGAC |
| **A1762T/G1764A SDM-FOR** | TGGGGGAGGAGATTAGGTTAA**T**G**A**TCTTTGTACTAGGAGG |
| **A1762T/G1764A SDM-REV** | CCTCCTAGTACAAAGA**T**C**A**TTAACCTAATCTCCTCCCCCA |
| **G1896A SDM-FOR** | CTGTGCCTTGGGTGGCTTT**A**GGGCATGGAC |
| **G1896A SDM-REV** | GTCCATGCCC**T**AAAGCCACCCAAGGCACAG |

**Table S2.** Primers used for qPCR for quantification of HBV DNA, cccDNA, and RNA as well as nested PCR of cccDNA and DIG-probe synthesis.

| **TARGET** | **FOR SEQUENCE (5’ TO 3’)*** | **REV SEQUENCE (5’ TO 3’)** | **ASSAY** |
| --- | --- | --- | --- |
| **HBV DNA** | CCGACCTTGAGGCGTACTTC | TACGGGTCAATGTCCATGCC | Supernatant HBV DNA qPCR |
| **HBV RNA 3’RACE** | ACCACGCTATCGCTACTCAC(dT)_17_GWAGCTC | N/A | cDNA synthesis of supernatant HBV RNA |
| **HBV RNA qPCR** | CAACTTTTTCACCTCTGCCTA | ACCACGCTATCGCTACTCAC | Quantification of supernatant HBV RNA |
| **cccDNA  (92F and 2251R)** | GCCTATTGATTGGAAAGTATGT | AGCTGAGGCGGTATCTA | HBV cccDNA qPCR |
| **HBxin** | ATGGCTGCTARGCTGTGCTGCCAA | AAGTGCACACGGTYYGGCAGAT | 3D-PCR |
| **cccDNA direct** | ACTCCTGGACTCTCAGCAATG | GTATGGTGAGGTGAGCAATG | Direct round of cccDNA PCR |
| **cccDNA nested** | AGGCTGTAGGCACAAATTGGT | CTTTATACGGGTCAATGTCCA | Nested round of cccDNA PCR |
| **X/BCP/preC** | GCATGGAGACCACCGTGAAC | CATAAGAGGACTCTTGGACT | DIG-probe synthesis |
| **preC/Core** | GCATGGAGACCACCGTGAACG | GAGGGAGTTCTTCTTCTAGG | DIG-probe synthesis |
| **Surface** | TGCTGCTATGCCTCATCTTC | CARAGACARAAGAAAATTGG | DIG-probe synthesis |

***D** = A, G, or T; **M** = A or C; **R** = A or G; **W** = A or T

**Table S3.** Primers used for qPCR for cellular mRNA analysis.

| **TARGET** | **FOR SEQUENCE (5’ TO 3’)*** | **REV SEQUENCE (5’ TO 3’)** | **ASSAY** |
| --- | --- | --- | --- |
| **GAPDH** | ACCAACTGCTTAGCCC | CCACGACGGACACATT | Cellular mRNA qPCR |
| **APOBEC3A** | GAGAAGGGACAAGCACATGG | TGGATCCATCAAGTGTCTGG | Cellular mRNA qPCR |
| **APOBEC3B** | GACCCTTTGGTCCTTCGAC | GCACAGCCCCAGGAGAAG | Cellular mRNA qPCR |
| **APOBEC3C** | CAACGATCGGAACGAAACTT | TATGTCGTCGCAGAACCAAG | Cellular mRNA qPCR |
| **APOBEC3DE** | ACCCAAACGTCAGTCGAATC | GCTCAGCCAAGAATTTGGTC | Cellular mRNA qPCR |
| **APOBEC3F** | CCGTTTGGACGCAAAGAT | CCAGGTGATCTGGAAACACTT | Cellular mRNA qPCR |
| **APOBEC3G** | GGTCAGAGGACGGCATGAGA | GCAGGACCCAGGTGTCATTG | Cellular mRNA qPCR |
| **APOBEC3H** | AGCTGTGGCCAGAAGCAC | CGGAATGTTTCGGCTGTT | Cellular mRNA qPCR |

**Table S4.** Detailed clinical and virological data of CHB carriers analyzed by next generation sequencing analysis.

| **AGE / SEX / ETH / GENOTYPE* (CASE ID#)** | **HBV DNA, IU/mL**^†^  (Date) | **qHBsAg, IU/mL** (Date) | **SERUM FULL-LENGTH / TRUNCATED HBV RNA, log copies/mL^‡^** | **ANTI- VIRAL THERAPY**** | **ALT** | **HBeAg/**  **Anti-HBe** (Date) | **PATHOLOGY / HCC THERAPY / FIBROSIS (FibroScan, kPa)**^††^ | **% OF HBV  QUASI-SPECIES^‡^** |
| --- | --- | --- | --- | --- | --- | --- | --- | --- |
|  |  |  |  |  |  |  |  | **A1762T / G1764A / G1896A** |
| **Pre-dominant wild-type** | | | | | | | | |
| 46 / F / CA / B  (30) | **1300 (2009)**  550 (2010)  2900 (2011)  870 (2012)  2200 (2013)  686 (2014)  1397 (2015)  1281 (2016)  825 (2017)  1275 (2018) | 2728 (7/2017) | 0 / 1.84 | NT | 11 | NEG/POS (03/2009) | LBx: (2009, 2013)  Grade 2; Stage 1  Stage 0 (4.9kPa) | 13.2 / 13.2 / 14.2  (2012) |
| 35 / M / AS / D  (83) | 9.9×10^5^ (2006)  **D (2012)**  D (2/2016)  8044 (7/2016)  9703 (5/2017)  U (6/2017)  D (6/2018)  425 (4/2019)  U (11/2019) | 1881 (2014) 1106 (2016) 860 (2017) 511 (2018) | 2.02 / 1.61 | TDF  (2006) | 23 | POS/NEG (2006)  NEG/POS (2013) | LBx: (2010)  Grade 1; Stage 2  Stage 0 (4.8kPa) | 21.5 / 21.3 / 13.7  (2012) |
| 48 / M / AS / C  (302) | 150 (01/2013)  12 (02/2014)  D (07/2014 to 01/2015) **D (07/2015)**  D (01/2016- 07/2016)  U (01/2017) | 387 (7/2014)  241  (7/2015)  91 (01/2016)  57 (08/2016)  49 (01/2017) | 3.12 / 2.50 | ETV  (2013-2015); TDF  (2015-present) | 38 | NEG/NEG (01/2013)  NEG/NEG (01/2017) | Cirrhosis, HCC:  RFA (2012);  SBRT (2014);  TACE (2015) | 16.1 / 15.9 / 16.1  (2015) |
| 38 / M / AF / E  (313) | 9.6×10^5^ (2013)  38 (10/2013  D (1/2014)  18 (04/2014)  56 (07/2014)  29 (10/2014)  U (01/2015)  D (04/2015 -2015)  **D (10/2015)**  D (04/2016 - 10/2016)  U (04/2017 -12/2019) | 770 (10/2014)  **270 (04/2016)**  247 (10/2016)  105 (04/2017)  86 (10/2017)  81 (05/2018) | 4.06 / 3.17 | TDF (2010-present) | 59 | NEG/POS (10/2016) | Cirrhosis, HCC  Resection (2014): Grade 3 poorly differentiated HCC; Stage 3 Fibrosis; Mild-moderate hepatitis  Resection (2015): Grade 3 poorly differentiated HCC; vascular invasion; Stage 3 Fibrosis; Mild-moderate hepatitis  Stage 4 (16 kPa, 2014);  Stage 2 (8 kPa, 2018) | 20.9 / 22.4 / 9.0  (2016) |
| **Pre-dominant G1896A** | | | | | | | | |
| 44 / F / AF / E  (47) | **4900 (11/2010)** 1100 (5/2011) | N/A | 2.47 / 1.95 | NT | 12 | NEG/POS (2008) | LBx: (2009) Grade 0, Stage 1 | 2.9 / 3.6 / 61.0 (2010) |
| 73 / F / AS / C  (266) | 1.2×10^6^ (04/2008)  1600 (07/2008)  110 (09/2008)  D (12/2008 -  06/2012)  33 (09/2012)  D (01/2013 - 09/2013)  72 (01/2014)  D (01/2014 -03/2014)  **U (10/2014)**  U (01/2015)  D (07/2015)  U (01/2016 - 10/2019) | **5.2**  **(01/2015)**  5.5  (10/15)  5.0 (07/2017)  4.5  (10/2017) | 3.11 / 1.71 | ETV  (2008-present) | 18 | POS/NEG (06/2007)  NEG/NEG (10/2019) | Cirrhosis, HCC  Resection (2014): Solid variant HCC; Severe fibrosis;  Mild hepatitis | 1.9 / 0.0 / 69.2  (2015) |
| 67 / M / AS / C  (CC15) | **U (10/2007)** | N/A | 0 / 3.59 | TDF | 84 | NEG/NEG | Cirrhosis, HCC:  Liver transplant | 0.5 / 0.7 / 98.3  (2007) |
| **Pre-dominant A1762T/G1764A** | | | | | | | | |
| 47 / M / AS / C  (6-2) | 2.3×10^6^ (02/2007)  1.9×10^6^ (9/2007)  **D (9/2009)**  D (03/2011  to 02/2014)  U (10/2014)  D (2/2015)  165 (8/2015)  U (12/2015 -11/2018)  D (06/2019)  U (03/2020) | 452 (10/2014)  515 (2/2015)  378 (03/2017)  309 (4/2018)  294 (06/2018) | 2.30 / 1.83 | (TDF)  ETV - present | 40 | NEG/POS (9/2010)  NEG/POS (06/2019) | LBx: (2007) Grade 0, Stage 1  Stage 0 (3.0 kPa, 2015);  Stage 0 (5.1 kPa, 2019) | 94.9 / 95.7 / 3.4  (2009) |
| 62 / M / AS / C  (113-2) | 3.6×10^7^ (4/2011)  890 (8/2011)  110 (11/2011)  23 (02/2012)  D (05/2012)  25 (8/2012)  40 (11/2012)  30 (02/2013)  D (05/2013 to 11/2016) **D (11/2017)**  U (5/2018)  D (12/2018) | 1288 (11/2014)  1140 (05/2015)  868 (5/2016)  **980 (11/2017)**  835 (12/2018) | 2.36 / 0.79 | TDF  (06/2011-present) | 28 | POS/POS (5/2011)  POS/NEG (11/2017) | Cirrhosis | 70.5 / 74.9 / 19.9  (2017) |
| 56 / M / AS / C  (307) | **5.5×10^5^** (09/2015)  U (06/2016)  13 (11/2016)  D (03/2017 -08/2017)  U (03/2018)  D (09/2018 - 03/2019)  U (11/2019) | **3231 (09/2015)**  1338 (08/2017)  599 (11/2019) | 2.51 / 2.90 | TDF  (09/2015-present) | 66 | NEG/POS (11/2016) | HCC  TACE (2015);  RFA (2016);  Left hepatectomy (2016)  Cirrhotic liver;  post-cautery changes; no HCC, partial gastrectomy erosion to gastric wall (11/2016)  Stage 1-2 (6.8 kPa, 2016) | 87.8 / 88.6 / 8.7  (2015) |
| 58 / M / AS / C  (405) | 2.6×10^6^ (11/2012)  365 (11/2014)  975 (03/2017)  106 (03/2018)  **73 (09/2018)**  D (01/2019)  U (12/2019) | 379 (03/2017)  21 (12/2019) | 0 / 4.59 | TDF  (2016 -present) | 19 | POS/POS (02/2007)  NEG/NEG (11/2014) | Resection  Moderately differentiated HCC, cirrhosis, no vascular invasion,  Stage 4 (16.8 kPa. 2015); Stage 4 (15.8 kPa, 2016) | 95.1 / 95.0 / 0.9  (2018) |

***AF**: African; **AS**: Asian; **CA**: Caucasian; **F**: Female; **M**: Male

^†^ Determined by clinical PCR assay (Roche TaqMan or Abbott Architect, LLOD:55, 20, or 10 IU/mL, respectively). **D**: Detectable but not quantifiable; **U**: Undetectable. Bolded dates are those within 6 months before sample collection.

**^‡^** Serum HBV RNA, genotype, and quasi-species proportions determined by RT-qPCR, phylogenic tree analysis, and NGS, respectively, in previous study (7,8)

****ETV**: Entecavir; **NT**: No treatment; **TDF**: Tenofovir

^††^Based on pathology, liver biopsy, or Fibroscan. **FS**: Fibroscan; **HCC**: Hepatocellular carcinoma; **Lbx**: Liver Biopsy; **RFA**: Radiofrequency ablation; **SBRT**: Stereotactic body radiation therapy; **TACE**: Trans-arterial chemoembolization
